# JAK2 46/1 (GGCC) Haplotype in Oncogenesis, as Risk Stratifier, and Indicator for Drug Resistance in Myeloproliferative Neoplasms

**DOI:** 10.3390/ijms262110337

**Published:** 2025-10-23

**Authors:** Michela Perrone, Sara Sergio, Beatrice Pranzo, Amalia Tarantino, Giuseppina Loglisci, Rosella Matera, Davide Seripa, Michele Maffia, Nicola Di Renzo

**Affiliations:** 1 Laboratory of Clinical Proteomic, “V. Fazzi” Hospital, 73100 Lecce, Italy; michela.perrone@unisalento.it (M.P.); beatrice.pranzo@unisalento.it (B.P.); 2 Laboratory of General and Human Physiology, Department of Experimental Medicine, University of Salento, 73100 Lecce, Italy; 3 Hematology Laboratory, Hematology and Stem Cell Transplant Unit, “V. Fazzi” Hospital, 73100 Lecce, Italy; amalia.tarantino@asl.lecce.it (A.T.); rosella.matera@asl.lecce.it (R.M.); davide.seripa@asl.lecce.it (D.S.); 4 Hematology and Stem Cell Transplant Unit, Onco-Hematological Department, “V. Fazzi” Hospital, 73100 Lecce, Italy; giuseppina.loglisci@asl.lecce.it (G.L.); nicola.direnzo@asl.lecce.it (N.D.R.)

**Keywords:** JAK V617F mutation, JAK2 haplotype GGCC_46/1, myeloproliferative, neoplasm, onco-drug resistance, risk stratification

## Abstract

The JAK2 46/1 (“GGCC”) haplotype is an inherited genetic variation within the *Jak*2 gene locus that has become a focal point in research related to oncogenesis, particularly in myeloproliferative neoplasms (MPNs). We conducted a narrative review of landmark discoveries in hematological malignancies and *Jak*2, focusing on its role in oncogenesis, risk stratification, and drug resistance in MPNs. This haplotype spans several polymorphisms within the *Jak*2 gene. It has been found to increase susceptibility to a variety of hematologic cancers, especially when linked with the somatic JAK2 V617F mutation, which results in the alteration of the JAK/STAT pathway, which is particularly essential for hematopoiesis. The “GGCC” part is characterized by four SNPs, with the G allele of the rs10974944 SNP in this haplotype correlated with MPNs progressing to myelofibrosis. Moreover, the G allele seems to be crucial for the predisposition to onco-drug resistance onset. To conclude, identifying the 46/1 haplotype in patients may not only enhance risk stratification for JAK2-driven cancers but also guide more effective, personalized therapeutic strategies to overcome resistance. Thus, this review aims to describe current knowledge about the JAK2 46/1 haplotype as a marker for diagnosis and the prediction of disease outcome.

## 1. Introduction

The JAK*2* 46/1 haplotype, also known as the GGCC haplotype, is an inherited genetic variation within the *Jak2* gene locus that has become a focal point in research related to oncogenesis, particularly in Myeloproliferative Neoplasms (MPNs) [1]. These hematologic malignancies, typified by clonal proliferation of myeloid lineage cells, include polycythemia vera (PV), essential thrombocythemia (ET), and primary myelofibrosis (PMF or MF), which are variably associated with mutations in the Janus Kinase 2 (*Jak2*) gene [2]. Central to their pathogenesis is the role of acquired somatic mutations, particularly the activating JAK2 V617F, which aberrantly activates the JAK/STAT signaling pathway—a key regulator of hematopoiesis, immune responses, and cellular growth [3].

Consequently, testing for *Jak2* mutations has become a standard component of evaluating patients with sustained high peripheral blood cell counts, organomegaly, unexplained thrombosis, or other symptoms indicative of MPN [4].

Notably, the 46/1 haplotype predisposes carriers to an increased risk of developing MPNs and is closely linked with the presence of the V617F mutation [5]. This haplotype spans several polymorphisms within the *Jak2* gene and has been found to increase susceptibility to a variety of hematologic cancers [6]. The “GGCC” part is characterized by four single-nucleotide polymorphisms (SNPs); it has been shown that the G allele of the rs10974944 single-nucleotide polymorphism of this haplotype is correlated with MPNs progression to MF and certain resistance-related clinical parameters [7]. Clinically, the 46/1 haplotype has emerged as a potential biomarker for risk stratification in MPNs, correlating with disease susceptibility, phenotype severity, and prognosis [7].

Identifying the 46/1 haplotype in patients may not only enhance risk stratification for *Jak2*-driven cancers but also guide more effective, personalized therapeutic strategies to overcome resistance.

Notably, another emerging research area relates to therapeutic challenges, focusing on the onco-drug resistance onset after the treatment with JAK inhibitors.

Thus, this review discusses the association between the haplotype and drug resistance mechanisms, emphasizing the urgent need for novel epigenetic and targeted approaches to address therapeutic refractoriness.

Moreover, by integrating foundational genetic insights with contemporary experimental and clinical findings, this review guides the reader through the evolving landscape of MPN biology shaped by the JAK2 46/1 haplotype. It highlights the haplotype’s multifaceted impact—from molecular pathogenesis to clinical management—while identifying critical gaps in knowledge and outlining future directions for research aimed at improving patient outcomes through precision medicine strategies.

## 2. Myeloproliferative Neoplasms

MPNs are clonal blood diseases characterized by the abnormal proliferation of bone marrow and blood cells, affecting precursors of white blood cells, red blood cells, and platelets to varying extents [8,9]. According to the 2022 ICC (International Consensus Classification) and World Health Organization (WHO) updates concerning the classification of hematopoietic neoplasms, MPNs are categorized into seven distinct disease types [10]. This classification encompasses:

(1) Chronic Myeloid Leukemia (CML), particularly the BCR:ABL1-positive subtype that can be further categorized into two phases: CML Accelerated Phase (CML-AP) and CML Blast Phase (CML-BP);
(2) Polycythemia Vera (PV);
(3) Essential Thrombocythemia (ET);
(4) Primary MyeloFibrosis (PMF), which is further divided into Early/pre-fibrotic primary myelofibrosis and Overt primary myelofibrosis.
(5) Chronic Neutrophilic Leukemia (CNL);
(6) Chronic Eosinophilic Leukemia (CEL);
(7) Myeloproliferative Neoplasm Unclassifiable (MPN-U) introduced to identify cases with morphological, clinical, or molecular features that do not fit the other definitions. MPN-U is often used for patients in the early diagnosis phase [11].

In 1951, William Dameshek coined the term “myeloproliferative disorders”, which was later renamed by the WHO “myeloproliferative neoplasms” [12]. They are typically differentiated from acute myeloid leukemia because they are characterized by fewer than 20% myeloblasts in the bone marrow and in the blood [13]. Additionally, differential diagnosis between MPNs and other non-acute myeloid neoplasms can be guided by the absence of significant morphologic dysplasia and characteristic patterns of hematopoiesis, as evidenced by elevated peripheral blood cell counts, splenomegaly, or both [14]. This contrasts with the ineffective hematopoiesis (characterized by a cellular bone marrow but reduced peripheral blood cell counts) and dysplasia seen in Myelodysplastic Syndromes (MDS) [11,15].

Hematopoietic stem cells (HSCs), produced in bone marrow, are immature cells with the ability to self-renew and differentiate in all mature blood cell types.

These cells can take two distinct paths at the progenitor stage, becoming myeloid or lymphoid stem cells. The first gives rise to T, B, and natural killer (NK) cells, while the second to megakaryocytes, erythrocytes, granulocytes, or macrophages [16]. The bone marrow environment, growth factors, and transcription factors are essential for a normal hematopoietic process. In MPNs, an abnormal proliferation of one or more terminal myeloid cell lines in the peripheral blood gives rise to the pathology. The myeloid lineage overproduces granulocytes, platelets, and erythrocytes [17]. This classification of MPNs is based on changes in blood cell counts and hematopoietic lineages in the bone marrow, which exhibit hyperplasia (an increase in the number of cells) and dysplasia (an abnormal development or growth of cells) [18].

In this paper, the considerations are focused on the three major MPNs.

PV is characterized by erythrocytosis, with morphologically normal cells. Patients with PV have higher thrombotic and hemorrhagic predisposition; moreover, there are cumulative risks of the evolution of the pathology into leukemia or risk for fibrotic progression [19].

The primary characteristics of ET are thrombocytosis in the peripheral blood and an increase in the number of mature megakaryocytes, the precursor cells of platelets, in the bone marrow. Thrombotic events account for more than 20% of the complications associated with ET, representing a major risk of this pathology. The aim of treatments available nowadays is to minimize the risk of thrombosis and/or bleeding [20].

Otherwise, MF refers to the formation of fibrosis in the bone marrow. MF can also derive from PV or ET [18]. The most characteristic features include bone marrow fibrosis (reticulin/collagen), aberrant inflammation with cytokine overexpression, anemia (resulting from ineffective erythropoiesis), hepatosplenomegaly, extramedullary hematopoiesis (EMH), constitutional symptoms (such as fatigue, night sweats, and fever), cachexia, leukemic progression, and shortened survival [21]. Approximately 20% of patients with PMF develop leukemia. Many others die from co-existing conditions, including cardiovascular complications and cytopenia-related issues like infections or bleeding [22,23,24].

Mutations in three key genes primarily drive these conditions: *Jak2*, Calreticulin (*Calr*), and Myeloproliferative Leukemia oncogene (*Mpl*). These mutations abnormally stimulate hematopoietic cell activity, leading to increased cellular proliferation and growth, which regrettably contributes to the progression of MF [18]. Therefore, one of these mutations is often associated with the pathology. For example, the *Jak2* mutation is found in approximately 90% of patients, making it a strong diagnostic indicator [21].

## 3. Role and Regulatory Mechanism of Wild Type JAK2

**Figure 1:**
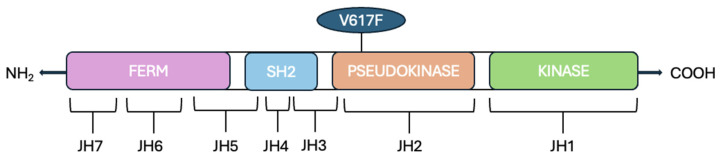
Schematic representation of the JAK2 (Janus Kinase 2) protein: it is composed of several functional domains. The N-terminal FERM domain encompasses the JAK homology (JH) regions JH7, JH6, and part of JH5. The adjacent Src Homology 2 (SH2)-like domain spans part of JH5, JH4, and most of JH3. The C-terminal region includes the pseudokinase domain (JH2), and the tyrosine kinase domain (JH1).

The *Jak2* gene is located on chromosome 9p24.1 that encodes a non-receptor tyrosine kinase. The *Jak2* gene comprises 142,939 base pairs (bp) and contains the following components: a promoter region, 25 exons, 25 introns, and a terminator region [25]. Alternative splicing produces three isoforms of the JAK2 protein, generating seven transcripts ranging from 6900 to 7000 bp [25]. In mammals, there are four members of the JAKs protein family: JAK1, JAK2, JAK3, and Tyrosine Kinase 2 (TYK2). JAKs proteins are composed of an N-terminal FERM (four-point-one, ezrin, radixin, moesin) domain, an Src Homology 2 (SH2) domain, a kinase-like pseudokinase domain (JH2) and a C-terminal tyrosine kinase domain JH1 [26] (Figure 1).

The FERM domain is composed of JAK Homology (JH) domains JH5, JH6, and JH7; this domain binds noncovalently to the juxtamembrane sequence of the cytokine receptor, which includes two segments [27,28], Box1 and Box2, respectively, characterized by proline-rich sequences and a hydrophobic segment. Moreover, this domain is involved in the intracellular regulation of JAK activity.

The SH2 domain lacks phosphotyrosine binding activity [26]. The name of the tyrosine kinase derives from the two kinase domains JH1 and JH2 because it refers to the double face of the Roman god Janus, a god of transitions or doorways. The first domain (JH1) encodes a Tyrosine Kinase (TK), while the second one (JH2) works as a Pseudokinase (PK) because it lacks many of the conserved residues that are essential for phosphotransferase activity [26].

## 4. JAK/STAT Pathway and V617F Mutation Involvement in MPN

JAK proteins are involved in the JAK/STAT pathway, an important signaling pathway downstream of cytokine receptors [29]. Cytokines are involved in numerous hematopoietic and immune functions, making this pathway essential for various cellular processes, particularly hematopoiesis [26,30]. Many different cytokines can activate the same JAK, which can be associated with multiple cytokine receptors, and in some cases, cytokine stimulation can enhance this association [29].

For instance, JAK2 associates with many homodimeric receptors, such as the Erythropoietin Receptor (EpoR), Thrombopoietin Receptor (TpoR/MPL), the Growth Hormone Receptor (GHR), and the heterodimeric receptors, including the Granulocyte-Macrophage Colony-Stimulating Factor (GM-CSF), and Interleukin-3 Receptors (IL-3R), which share a common beta subunit (βc) [31].

*Jak2* is highly expressed in hematopoietic cells and is critical for hematopoiesis, like *Jak3*, which is especially expressed in lymphoid lineage cells [26]. Instead, *Tyk2* is also detected in non-hematopoietic tissues, including the liver, lung, and intestine, where it participates in immune and inflammatory responses.

In the JAK/STAT pathway the binding of the hormone, Interferons (IFNs), Interleukins (ILs), Colony-Stimulating Factors (CSF), cytokines, and growth factors lead to receptor dimerization [32].

Ligand binding activates the phosphorylation of tyrosine residues in the cytoplasmic domain of the receptor and in JAK2 itself. It induces receptor rearrangement that allows the transphosphorylation of the activation loop located on the JH1 domain, causing JAK2 activation [33]. There are approximately 20 residues of tyrosine involved in this phosphorylation, which originates from cytokine activation [34].

The activation loop is the part in which the major autophosphorylation of the JAK protein, a prerequisite for catalytic activation, occurs. This site includes all JAK tandem tyrosine residues; for instance, in JAK2, there are Tyr^1007^ and Tyr^1008^ [35]. Moreover, other Tyr residues phosphorylated could enhance or downregulate JAK2 activity. The activation mechanism by Tyr^1007^–Tyr^1008^ phosphorylation is not yet fully understood in all cases; for instance, it has been demonstrated that phosphorylation of Tyr^119^ in the FERM domain of JAK2 regulates its association with the Epo receptor [36,37]. Otherwise, in the absence of cytokine activation, JAK2 is phosphorylated on Ser^523^, which negatively controls JAK2 activity [38].

After activation, the docking site becomes available and can attach signaling proteins, such as STAT (Signal Transducer and Activator of Transcription). Two STAT proteins, phosphorylated on a conserved tyrosine residue at the C terminus, dimerize, dissociate from the receptor, and go to the nucleus, where they regulate gene expression; in fact, they bind DNA and activate the transcription of the target gene [3]. Their binding site is typically located in specific regions, such as the enhancer, promoter region, or first intron of the target gene [3].

Considering that JAK/STAT pathway is fundamental in hematopoiesis [26], its alteration leads to many blood diseases. A genetic polymorphism of *Jak2*, the mutation V617F, has been associated with MPNs [39]. It occurs in nearly 95% of patients with PV, and in 50–60% of patients with ET and PMF [30].

JAK2 V617F is a somatic mutation, not present in the germline DNA of patients, acquired in the hematopoietic compartment, and characteristic of hematopoietic cells [32]. This mutation consists of the substitution of valine for phenylalanine at codon 617 of JAK2, resulting in a gain-of-function effect for this protein [6,25,32].

There are three main hypotheses about the physiopathological mechanism after the mutation.

The first current of thought states that constitutive activation of the pseudokinase (JH2) domain drives to loss of inhibitory function that it exercises over the active (JH1) kinase domain. The roles of the JH2 domain, in fact, are to maintain a low basal activity of JAK when there are no cytokines and to facilitate JAK activation upon binding to the receptor when cytokines are present [40].

The second hypothesis concerns the possibility that changes in JH2 conformation are responsible for kinase domain activation. The V617 mutation, which is mutated to Phe, could influence the neighboring F595, a residue positioned in the αC helix of JH2, in a specific manner. The specific conformation of the αC helix is fundamental for kinase domain activation [41].

The last hypothesis focuses on the direct activation of the JH1 domain via an SH2-JH2 linker [42]. V617F is one of three mutations known to be concentrated in 3 regions of JH2; V617F is encoded by exon 14 and it is present in the majority of MPNs, while mutation on the exon 16 is associated with B-cell leukemia and in 4% of PV cases it has been found a mutation in the linker between SH2 and JH2 domain, encoded by exon 12 [40]. These findings demonstrate the importance of the JH2 domain in regulating this pathway.

**Figure 2:**
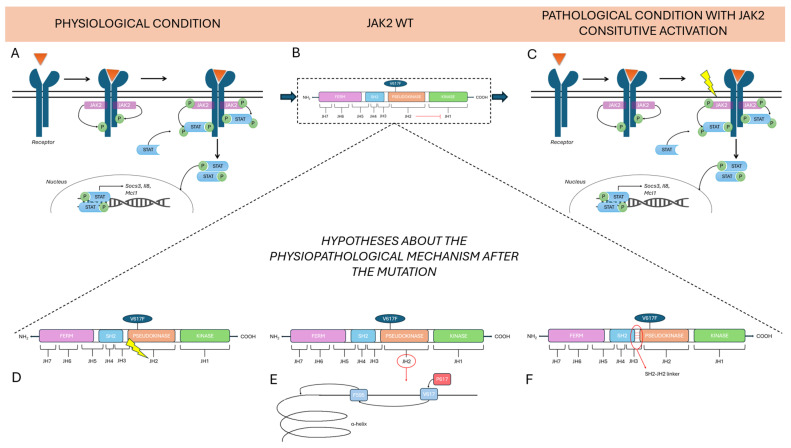
Physiological and pathological conditions mediated by JAK2 signaling and hypotheses on the role of the V617F mutation. The figure compares the physiological condition (**A**) with the pathological one (**C**). In panel (**B**) is represented the wild-type JAK2, characterized by the inhibitory activity of JAK2 pseudokinase (JH2 domain) on JH1. (**A**) In normal conditions, upon ligand (in the figure represented by the orange triangle) binding to the transmembrane receptor, this undergoes dimerization, activating the associated tyrosine kinase JAK2. Activated JAK2 phosphorylates specific tyrosine residues on the receptor, generating docking sites for STAT proteins. Once recruited, STATs are subsequently phosphorylated by JAK2, dimerize, and translocate into the nucleus, where they regulate the transcription of target genes like *Socs3*, *Il8* and *Mcl1*. (**C**) V617F mutation in the JAK2 JH2 domain induces constitutive activation of the JAK/STAT pathway regardless of the presence of the ligand. The pathogenic JAK2 constitutive activation is typical of some MPNs. Pathogenetic hypotheses include the functional impairment of the JH2 domain leading to a constitutive activation of JAK2 even in the absence of external stimuli (**D**), an alteration of the interaction with the F595 residue, which is located in the αC helix of the JH2 domain, which alters the helix’s structure (**E**), and an abnormal interaction between the SH2 and JH2 domains in the SH2-JH2 linker (**F**).

In any case, the mutation in the JAK2 causes an alteration in the stability of the protein, which leads to the constitutional activation of the tyrosine kinase, with the subsequent downstream activation of STAT factors (Figure 2).

Otherwise, STAT proteins are signal transducers and activators of transcription. STAT family includes seven members: STAT1, STAT2, STAT3, STAT4, STAT5a, STAT5b, and STAT6. Their primary function is as transcription factors, so when they enter the nucleus, they regulate the expression of cytokine-responsive genes [43].

Mainly STAT3 and STAT5 are activated upon the mutation of JAK2. For instance, constitutive activation of STAT5 has been associated with leukemic transformation [44]. Moreover, high levels of STAT5 and STAT3 phosphorylation have been observed in PV patients, while in ET patients, STAT3 is highly phosphorylated, whereas STAT5 exhibits decreased phosphorylation levels. Ultimately, in PMF patients, both STAT3 and STAT5 exhibit low phosphorylation levels. Thus, in this cascade, a specific cytokine activates a specific JAK, which in turn activates a specific STAT. Therefore, some proteins dampen cytokine signaling at the different levels of this pathway. For instance, the SOCS (Suppressors Of Cytokine Signaling) family inhibits this cascade through negative feedback [45]. For example, SOCS1-7 and the Cytokine-Inducible SH2-containing protein (CIS) act by recruiting ubiquitin ligases (E3) to target JAK2 for degradation, whereas SOCS3 directly inhibits JAK2’s kinase activity via complex formation [4].

Thus, when V617F mutation is present, hematopoietic cells undergo transformation into cytokine-independent growth. Therefore, these altered processes lead to adverse effects like tumorigenesis, tumor progression, and the resulting inflammation [25,46].

## 5. JAK2 GGCC 46/1 Haplotype Discovery and Pathophysiology

Recent findings have shown that JAK2 V617F is associated with this specific haplotype, the germline GGCC (46/1) haplotype, especially in MPNs [25,46]. Haplotype 46/1 was first discovered by Jones et al. [47]. They found out that 77% of cases (109 patients) share the identical haplotype, subsequently designed as 46/1 within the *Jak2* gene [47].

A haplotype is a group of genetic variations present on the same chromosome. These variants are not easily separable by recombination and tend to be inherited together as a unit. They are in linkage disequilibrium, which is proof of a shared ancestry of the chromosomes.

These results indicate that the homozygous V617F mutation is not random, in fact it occurs on a specific JAK2 haplotype. This haplotype is present in approximately 45% of the general population [25], and other studies have confirmed its association with the V617F mutation [25,46].

The 46/1 haplotype contains a segment called “GGCC” that encompasses the most frequently mutated *Jak2* exons: exon 14 (mainly the V617F mutation), exon 12 (mutations and deletions), and, to a lesser extent, exons 13 and 15 [25].

This haplotype includes a set of genetic variations that map a region of 250–280 kb and are distributed along chromosome 9p.24.1, which covers the *Jak2*, *Insulin-Like 6* (*Insl6*), and *Insulin-Like 4* (*Insl4*) genes [5,25,46]. The last two genes are generally not transcribed in the hematopoietic system [48].

Genetic variations within the considered region are located on sites of single-base, thus they are known as Single-Nucleotide Variants (SNVs). These SNVs are crucial for inherited and interindividual variation observed in complex phenotypes [24].

**Figure 3:**
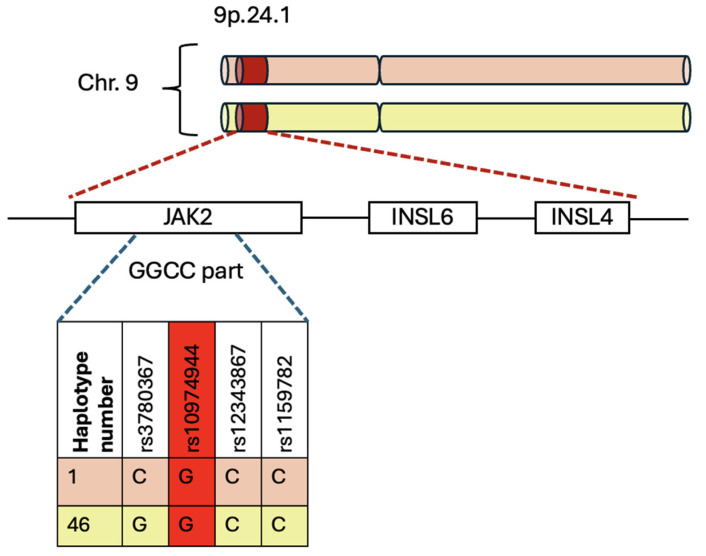
Schematic representation of 46/1 haplotype’s localization on chromosome 9p.24.1: In position 9.24.1 there is 46/1 haplotype, which encompasses three genes: *Jak2*, *Insl6* and *Insl4*. The GGCC part is a specific region of this haplotype, which is tagged by 4 SNPs. The red section highlights the SNP discussed in the text.

The “GGCC” part encompasses the region between intron 10 and intron 15 of the *Jak2* gene, characterized by four Single-Nucleotide Polymorphisms (SNPs): rs3780367 in intron 10, rs10974944 in intron 12, rs12343867 in intron 14, and rs1159782 in intron 15 [49]. All these SNPs are in complete linkage disequilibrium, which means they are inherited in tandem. The term “GGCC” is not derived from its occurrence in GC sequences, but rather from the fact that it contains four single-nucleotide polymorphisms (SNPs) [5]. These SNPs replace three thymidines (T) with two guanosines (G) and one cytosine (C) with another cytosine, resulting in the distinctive pattern “GGCC” (Figure 3) [25].

A novel hypothesis associates the GGCC haplotype with dysregulation in inflammatory and myelomonocytic responses, possibly due to the increased expression of the other two genes covered by the haplotype, *Insl6* and *Insl4* [25]. They increase their expression in the medullary stromal cells, where they are produced; consequently, these cells produce excessive amounts of proinflammatory cytokines [49].

In addition to inflammation, other events such as splenomegaly and splanchnic vein thrombosis have been associated with 46/1 [25,46].

Another influence of the haplotype is exercised on the expression of *Insl6* and *Insl4* in medullary stromal cells, causing proinflammatory and promyeloid activity of cytokines. This mechanism facilitates the survival of the mutated clone [50].

Interestingly, several individuals in an Australian family with familial MPN, identified with germline Retinoblastoma-binding protein 6 (*Rbbp6*) variants, also carried the JAK*2* 46/1 haplotype [51]. This suggests these germline predisposing variants may act additively or synergistically to promote MPN development. It means that the presence of each variant independently contributes to the overall risk of developing MPN or that their combined effect is greater than the sum of their individual effects.

*Jak2* expression appears to be unaffected by the 46/1 haplotype [1].

Anyway, the JAK*2* GGCC_46/1 haplotype is significantly more prevalent in individuals with the JAK2 V617F mutation. Its frequency, approximately 24% in the general population, rises considerably between 40% and 80% in patients who have the V617F mutation [25]. So, JAK*2* haplotype GGCC_46/1 is preferentially associated with JAK2 V617F mutation, but not exclusively [5]. This haplotype, which accounts for approximately 28% of the population-attributable risk of developing a myeloproliferative neoplasm (MPN), precedes the acquisition of the JAK2 V617F variant [25]. It has also been identified as a significant factor that increases the risk of familial MPNs by more than five times as shown by different studies in populations from Brazil, Japan, and China [46,52].

The “GGCC” haplotype is associated with rs10974944, a single-nucleotide polymorphism (SNP) where a cytosine is replaced with a guanosine (Figure 3). Consequently, the C allele is the prevalent allele, while the G allele signifies the variant that increases the risk of myeloproliferative neoplasms (MPNs). Whereas the heterozygous condition is characterized by the presence of both C and G alleles [25].

The study carried out by Kilpivaara, which identified the rs10974944 variant (C/G) in the *Jak2* gene, have demonstrated that the rs10974944 (G) allele may predispose to the acquisition of the JAK2 V617F somatic variant on the same strand [25,53]. In addition, the C-allele has been linked to ET. Conversely, other studies have shown a prevalence of the heterozygous haplotype CG in patients with PMF and PV [54].

Therefore, the JAK2 46/1 haplotype in patients with MPNs could potentially serve as a biomarker for the early diagnosis of the condition.

## 6. JAK2 Haplotype 46/1 and Onco-Drug Resistance Onset

### 6.1. MPN Treatments and Therapies

MPNs are typically slow progressing chronic disorders [55]. In the management of MPNs, treatment goals must be clearly tailored to the individual patient’s clinical presentation, risk profile, and disease subtype. The primary objectives of therapy include the following:

(1) Prevention of disease-related complications: MPNs are associated with a high risk of thromboembolic and hemorrhagic events, particularly in PV and ET. Indeed, treatment strategies aim to minimize these risks through cytoreductive therapy, antiplatelet agents, and phlebotomy, depending on patient-specific factors [56].
(2) Risk-adapted therapeutic approaches: Therapeutic decisions should be guided by established prognostic models (e.g., International Prognostic Score for ET (IPSET), Dynamic International Prognostic Scoring System (DIPSS)) and take into account age, symptom burden, cardiovascular risk factors, *Jak2/Mpl/Calr* mutation status, and previous thrombotic events. Low-risk patients may benefit from observation or minimal intervention, whereas high-risk individuals may require more aggressive cytoreductive therapy [57].
(3) Prevention of disease progression and leukemic transformation: Although many MPNs follow a relatively less aggressive clinical course, it remains a significant risk of progression to MF or acute myeloid leukemia (AML), particularly in PMF and in long-standing cases of PV or ET. Long-term management aims to delay or prevent such evolution through appropriate monitoring and timely therapeutic escalation [57].
(4) Achievement of disease modification or cure: While a definitive cure remains elusive for most MPNs, certain disease-modifying agents have shown potential for deep molecular and clinical responses. Interferon-alpha, particularly in early-stage PV or ET, has demonstrated the ability to induce hematologic remission, reduce JAK2 V617F allele burden, and possibly alter the natural course of the disease. Allogeneic stem cell transplantation may offer a curative approach in selected high-risk patients with PMF or post-MPN AML [58,59].

Indeed, nowadays, the therapies employed in MPNs treatment include: (i) cytoreductive therapy, used to reduce elevated blood counts and lower the risk of thrombosis; (ii) JAK inhibitors, targeting the JAK-STAT pathway, particularly in patients with splenomegaly and systemic symptoms; iii) antiplatelet or anticoagulant therapy to reduce thrombotic events; (iv) phlebotomy, preferentially used in PV to maintain hematocrit <45% in men and <42% in women; (v) Allogeneic Hematopoietic Stem Cell Transplantation (HSCT), the only curative treatment, primarily reserved for high-risk or advanced myelofibrosis, or MPNs progressing to acute leukemia, which due to high risk, is generally used in younger patients or those with poor prognosis; and (vi) Symptom Management (Antihistamines or supportive care—Blood transfusions, iron chelation, and erythropoiesis-stimulating agents in anemia).

Among cytoreductive therapies, Oncocarbide, also known as hydroxyurea (ATC: L01XX05), is an antimetabolite that acts through the inhibition of DNA synthesis, according to the National Cancer Institute definition. So, it exerts cytostatic activity. It is a ribonucleotide reductase inhibitor that does not interfere with RNA or protein synthesis [60].

Recent progress in understanding cytokine signaling and intracellular pathways has led to the development of JAK inhibitors, which target the JAK-STAT pathway, frequently activated in MPNs due to mutations such as JAK2 V617F [61]. The development of targeted therapies was strongly supported by two key findings: gene knock-out studies and the clinical discovery of activating JAK mutant forms (e.g., the JAK2 V617F mutation in myeloproliferative disorders). This molecular evidence clearly establishes that aberrantly activated JAK signaling is the central pathogenetic driver [62].

Among JAK inhibitor, one of the most used drugs is Ruxolotinib, a selective inhibitor of JAK1 and JAK2 [63]. This type of drug acts on the JAK-STAT pathway, reducing the phosphorylation and leading to reduced cellular proliferation and induction of apoptosis [64]. Ruxolitinib is particularly indicated for the treatment of splenomegaly and other symptoms in adults with PMF, or MF that develops after PV or ET [65]. Otherwise, Fedratinib (TG101348) is a selective JAK2 inhibitor that can induce apoptosis in cell lines with the V617F mutation [66,67]. They are both “type I” inhibitors, which means that they bind and stabilize the kinase-active conformation of JAK2. In contrast, “type II” ATP-competitive inhibitors do the same but with the inactive conformation of the protein [68].

**Figure 4:**
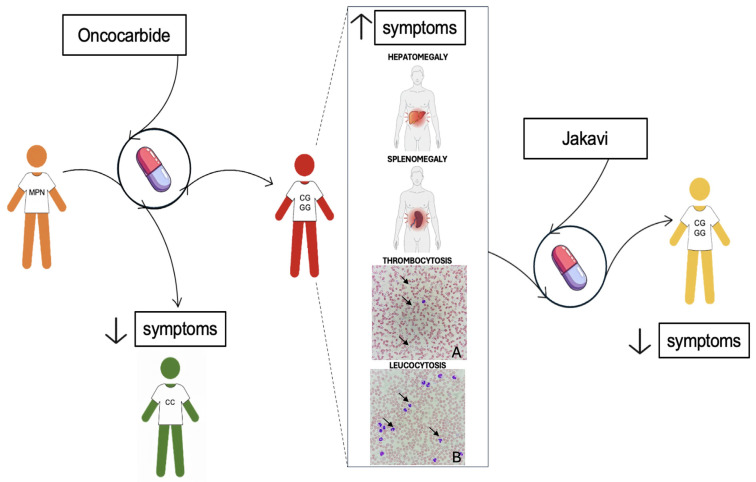
Schematic representation of 46/1 haplotype correlation with symptoms related to onco-drug resistance onset in MPN patients: Patient diagnosed for MPN (in orange) undergoes treatment with Oncocarbide as first-line therapy. CC haplotype (in green) correlates with good disease outcome, with improvement of symptoms (downward arrow). Otherwise, CG or GG conditions (in red) are more often associated with patients’ worsening, leading to the onset of the symptoms (upperward arrow) related with the onco-drug resistance, such as hepatomegaly, splenomegaly, thrombocytosis and leukocytosis. In the last case, ruxolitinib is used as second-line therapy (in yellow) for the improvement of symptoms (downward arrow). Microscopic images of thrombocytosis and leukocytosis were acquired using a 100× oil immersion objective. Black arrows show platelets in A and leucocytes in B.

Recommendations from the National Comprehensive Cancer Network (NCCN) [69] and European LeukemiaNet (ELN) [11] offer disease-specific treatment strategies:

(1) Regarding PV, hydroxyurea (Oncocarbide) serves as the primary treatment for high-risk patients [70]. In contrast, it is used for low-risk patients when blood counts rise, symptoms intensify, or splenomegaly or phlebotomy intolerance occurs [15]. If hematocrit surpasses 45%, the therapy switches to ruxolitinib, which is pivotal in managing hematocrit and reducing splenomegaly [71,72].
(2) Oncocarbide is also used as a first-line treatment in patients with essential thrombocythemia (ET). If hematocrit levels rise or the patient develops leukocytosis or thrombocytosis during treatment, the cytoreductive therapy is switched to ruxolitinib. For low-risk ET patients—typically younger individuals without additional risk factors or extreme thrombocytosis—management may consist of low-dose aspirin or simple observation [73]. However, extreme thrombocytosis (platelet count > 1 × 10^6^/μL) poses a bleeding risk and requires closer monitoring [15]. High-risk patients are usually treated with cytoreductive therapy, with hydroxyurea as the standard first-line option. In cases of intolerance or resistance to hydroxyurea, alternatives such as anagrelide or ruxolitinib may be considered. Regardless of risk category, all patients should undergo careful management of cardiovascular risk factors [73]. In ET, Oncocarbide can be prescribed at doses up to 1.5–2 g per day to reduce platelet counts [74]. If platelet levels continue to rise despite treatment, a change in therapy is necessary [75]. Splenomegaly is another important clinical parameter; an increase in spleen size during Oncocarbide therapy may indicate developing resistance to the drug [11].
(3) For MF patients, hydroxyurea is the first-line treatment, effectively halving splenomegaly in about 40% of cases, with benefits lasting roughly one year [76]. Myelosuppression and painful mucocutaneous ulcers are common side effects. If patients become refractory to hydroxyurea (e.g., hematocrit above 45%) [11] or experience symptomatic splenomegaly, treatment should switch to ruxolitinib [63] (Figure 4).

So, although many patients with MF have shown some improvement in the signs and symptoms after treatment with ruxolitinib, others are refractory to ruxolitinib, and a lot of them lose their response over time. Nevertheless, a possible prolongation of patients’ survival has been observed in other cases [77].

### 6.2. Challenges and Drug Resistance in MPNs

The two considerable situations to evaluate for changing therapy are drug resistance and drug intolerance. The first manifests as splenomegaly, leukocytosis, thrombocytosis, or persistent anemia; hematological clinical signs, such as anemia, or extra-hematological clinical signs, including non-melanoma skin cancers, characterize the second [11].

Drug resistance in JAK2-associated cancers, particularly to JAK inhibitors like ruxolitinib, is a significant challenge in treating MPNs. The 46/1 haplotype has been implicated in influencing responses to these drugs and the development of resistance, and various mechanisms have been hypothesized to explain this association.

The first hypothesis concerns an enhanced activation of signaling pathways. It has been demonstrated that if the haplotype enhances the V617F mutation, this alteration may lead to more robust activation of downstream signaling pathways, such as JAK-STAT [47]. However, it can also increase Mitogen-Activated Protein Kinase (MAPK) activity, which plays a key role in promoting cell growth and survival. In this signaling network, MAPK activation, induced by JAK2 V617F, is, to some extent, also dependent on PhosphatidylInositol 3-Kinase (PI3K) [61]. Thus, because the resilience of the JAK2 and PI3K pathways may reduce the efficacy of JAK inhibitors, these pathways could be a potential therapeutic target for controlling abnormal cell growth in JAK2 V617F-positive diseases, potentially with alternative or combinatorial approaches [78]. A possibility is to target both the JAK/STAT and Bromodomain and Extra-Terminal motif (BET) pathways to improve response rates. The BET family comprises four proteins, BRD2, BRD3, BRD4, and BRDT, which are a group of transcriptional co-regulators [79]. They can recognize acetylated lysines in histones [79]. The BET inhibitors prevent them from binding these acetylated residues [80]. BET proteins and JAK2 signaling can converge on the same transcriptional regulatory system, a key point in controlling the expression of specific genes, such as LIM domain only 2 (LMO2), which is expressed in progenitor cells from PV patients [81]. It has been discovered that the combined action of two inhibitors may prevent the rise in JAK inhibitor-resistant clones. For instance, I-BET151 is effective against PV, as the compound specifically targets homozygous JAK2 V617F cells. In polycythemia, both JAK and BET inhibitors, which do not induce a consistent reduction in the burden of JAK2 V617F-positive progenitor cells [82], may overcome the problem of the first one and may increase the possibility of targeting the neoplastic cells while simultaneously reducing the risk of adverse side effects.

In any case, substantial evidence suggests that the resistance to the JAK inhibitor may be a consequence of the reactivation of the JAK/STAT pathway through the heterodimerization of activated JAK2 and JAK1, or TYK2 [83]. This mechanism results in the activation of JAK2 in trans by other JAK kinases, increased JAK2 mRNA expression (which promotes the formation of heterodimers), and, ultimately, the resistance to JAK2 inhibitor-induced apoptosis [83]. In these studies, the cessation of therapy with a JAK2 inhibitor results in new sensitization of the resistant clone positive for the V617F mutation, leading to the conclusion that after a period of treatment interruption, patients resistant to JAK2 inhibitors may respond to retreatment with the same JAK2 inhibitor or with others [84].

Another hypothesis concerns the altered epigenetic landscape, as it has been confirmed that the V617F mutation induces epigenetic deregulation [83]. Epigenetic remodeling is essential in hematopoietic cells because it ensures their steady state, defined as the equilibrium between self-renewal and proliferative gene expression programs, thereby guaranteeing the appropriate cell fate determination [85]. Reversible modifications to chromatin structure, DNA methylation, and histone modifications are all epigenetic changes that influence gene transcription profiles [86].

It has been demonstrated that JAK inhibition leads to alterations in the histone landscape, resulting in an increase in methylation and acetylation [87]. Alteration of the methylation landscape has been suggested to play a key role in the pathogenesis and leukemic transformation of MPNs [88].

To conclude, the 46/1 haplotype might affect gene expression patterns and methylation status in hematopoietic stem cells, promoting a survival advantage. This epigenetic advantage enables cells to bypass JAK inhibition, allowing them to adapt to the selective pressure exerted by JAK-targeted therapies [83]. Indeed, several studies have suggested that the haplotype is associated with an increase in the expression of specific genes, including *Jak2*, *Insl6*, and *Insl4* [49]. Therefore, this process results in DNA recombination, with the emergence of genetic variations or abnormal methylation of the promoter region too [25]. For instance, DNA recombination could be stimulated by unidentified intronic repeating DNA sequences in the 46/1 haplotype, which could, in turn, cause the overexpression of the *Jak2* gene located on the recombined allele [25]. The activation of *Jak2* determines the transmission of signals derived from all the cytokines involved in myelopoiesis, resulting in the chronically excessive stimulation of this process [61]. In conclusion, the excessive stimulation of mitosis in myeloid progenitors makes them more prone to making mistakes during replication, creating altered genes, such as *Jak2* [25].

Another hypothesis regarding the influence of the 46/1 haplotype on drug response and resistance suggests that cells carrying this haplotype may be more prone to acquiring additional mutations during treatment [83]. This increased mutational susceptibility could lead to the emergence of drug-resistant clones. Such a mechanism is particularly concerning in MPNs, where clonal evolution plays a key role in both disease progression and resistance to therapy. The JAK2 V617F allele burden, also referred to as the variant allele fraction (VAF), indicates the proportion of hematopoietic cells harboring the mutation [46]. A low clonal burden of JAK2 V617F is typically associated with phenotypes such as clonal hematopoiesis of indeterminate potential or essential thrombocythemia (ET), while a high clonal burden is more often linked to polycythemia vera (PV) or post-PV myelofibrosis (MF) [70]. Elevated JAK2 V617F VAFs are correlated with increased leukocyte counts, higher risk of transformation to myelofibrosis, and a greater likelihood of developing splenomegaly [48]. These findings support the idea that hematopoietic cells carrying the JAK2 V617F mutation—frequently found in individuals with the 46/1 haplotype—undergo more aggressive clonal evolution [89]. This evolutionary process not only contributes to disease progression but also to the development of resistance to treatment, a defining feature of MPNs, especially in advanced stages such as myelofibrosis [90].

Moreover, while direct evidence linking the 46/1 haplotype to drug resistance is limited, it has been associated with clinical markers of resistance and disease transformation, such as progression to MF. In one study, the G/G allele was observed in patients with resistance-related parameters (*p* = 0.002449) [7].

The JAK*2* 46/1 haplotype is a significant factor in both the development and therapeutic resistance of JAK2-associated hematologic cancers. Its association with the JAK2 V617F mutation highlights its role in cancer susceptibility, while its impact on signaling pathways and the epigenetic landscape of cells contributes to the development of drug resistance. Identifying the 46/1 haplotype in patients may not only enhance risk stratification for JAK2-driven cancers but also guide more effective, personalized therapeutic strategies to overcome resistance. Further research into its exact mechanisms may reveal additional targets for intervention, offering new hope for treating resistant hematologic malignancies.

## 7. JAK2 Haplotype 46/1 and Risk Stratification

A recent study has revealed that the 46/1 haplotype, specifically the G allele of the rs10974944 SNP, is associated with several clinical features related to the onset of onco-drug resistance. In this study, researchers selected 50 patients from the 665 who underwent analysis for the JAK2 V617F mutation. Only patients who tested positive and had a specific diagnosis of one of the three MPNs considered were deemed suitable for further analysis. Among them, 19 have polycythemia vera, 18 were diagnosed with primary myelofibrosis, and 13 have essential thrombocythemia. In particular, 18 of them present the homozygous common condition for the 46/1 haplotype, the CC allele, 29 the heterozygous allele (CG), and only 3 have the G allele [7].

In fact, the rs10974944 G allele, a tag for the JAK2 46/1 haplotype, has been shown to be significantly more prevalent in JAK2 V617F-positive patients, suggesting a germline predisposition that affects disease phenotype and clinical progression [52]. The clinical parameters considered to be associated with the onco-drug resistance onset are splenomegaly, hepatomegaly, thrombocytosis and leukocytosis [7]. A Brazilian study found that MPN patients carrying the G allele exhibited higher hematocrit, hemoglobin, and platelet levels compared to non-carriers, correlating with clinical manifestations like splenomegaly and thrombocytosis [91].

A recent cohort study of drug-resistant MPN patients from Southern Italy identified the C/G genotype in approximately 58% of resistant individuals, who frequently displayed hepatomegaly, thrombocytosis, and leukocytosis, while the G/G genotype was underrepresented (~6%) and not significantly associated with resistance outcomes [7].

Nonetheless, the presence of the G/G genotype appears to be related to a clinical profile (e.g., splenomegaly and high blood cell counts) that could potentially favor the development of resistance-related conditions, possibly through chronic inflammation and clonal evolution mechanisms (Figure 4).

Importantly, large cohort studies have provided quantitative evidence supporting the role of this haplotype in early disease risk stratification. One large cohort study reported an Odds Ratio (OR) of 2.8 for JAK2 V617F positivity among individuals with the G allele compared to non-carriers [89]. Additionally, the GG/CC genotype at rs10974944 contributes significantly to familial MPN risk, with an OR of 2.80 and a Population Attributable Risk (PAR) of 46.0%, reflecting a substantial impact of this variant on disease predisposition at the population level [92]. While familial relative risks attributed to this variant are smaller (e.g., <3% for Chronic Lymphatic Leukemia (CLL)), the genotypic relative risks reported for JAK2-positive MPNs are among the highest described in genome-wide association studies [92].

More compelling, this haplotype—specifically the G allele of rs10974944—has also been associated with an increased risk of progression to MF, a more severe MPN phenotype. Several studies indicate that 46/1 carriers are more likely to harbor not only JAK2 mutations but also MPL or CALR mutations, increasing the risk of fibrotic transformation and leukemic progression [6,70,93]. Moreover, recent insights from 3D chromatin structure studies suggest that the 46/1 haplotype may promote a Programmed Death-1 receptor Ligand (PD-L1)–mediated immunosuppressive niche, contributing to disease progression and treatment resistance [94].

Despite promising data, the 46/1 haplotype has yet to be fully integrated into standardized risk assessment models in current clinical practice. Indeed, the 46/1 haplotype has not yet been integrated into standard risk assessment tools such as the International Prognostic Scoring System (IPSS) or Mutation-Enhanced IPSS for patients <70 (MIPSS70) [55]. These tools primarily incorporate clinical and somatic mutation data (e.g., *Jak2*, *Calr*, *Mpl*, *Asxl1*, *Srsf2*) to guide prognosis and therapeutic decisions. However, emerging evidence suggests that germline predisposition, particularly involving the 46/1 haplotype, may refine these models further—especially for predicting disease progression to MF or treatment resistance. Given that 46/1 carriers often exhibit a proinflammatory and proliferative phenotype, incorporating germline markers like rs10974944 into existing models could enable more personalized risk stratification, identifying patients who might benefit from earlier intervention, closer monitoring or inclusion in clinical trials exploring disease-modifying therapies.

In summary, while current prognostic systems do not account for germline variants such as the 46/1 haplotype, its strong association with disease phenotype, mutation burden, and progression risk supports its potential integration into next-generation risk stratification algorithms for MPNs (Table 1).

## 8. Last Frontier: Epigenetic Therapies

Epigenetic therapies, such as DNA Methyltransferase (DNMT) or Histone DeACetylase (HDAC) inhibitors, could be investigated as adjuncts to standard JAK inhibition in patients with the 46/1 haplotype, targeting the modified epigenetic landscape that might confer a survival advantage. DNMT and HDAC inhibitors induce DNA demethylation and histone acetylation, respectively, leading to the reactivation of silenced genes and dramatic morphological and functional changes in cancer cells [95]. One of the consequences of HDAC inhibition is the continuous activation of the STAT protein, involved in the JAK/STAT pathway [96]. For example, HDAC8 inhibitors modulate the JAK/STAT pathway in patients with MPN [97]. It has been demonstrated that HDACi, when combined with JAK2i (JAK2 Inhibitor), inhibits the growth of breast cancer cells, leading to apoptosis [98].

Otherwise, DNMT can act on the JAK7STAT3 pathway, for instance, reducing its activation. DNMT inhibitors reverse DNA hypermethylation, which may lead to the reactivation of silenced tumor suppressor genes or negative regulators of the JAK/STAT pathway [99]. For example, they can act on genes encoding SOCS proteins, which are key negative regulators of the JAK/STAT pathway. If these genes are epigenetically silenced in MPNs, DNMT inhibitors can restore their expression and dampen pathway activation [100].

So, although JAK inhibitors primarily reduce symptoms, control splenomegaly, and improve quality of life, they do not fully eradicate the mutant clone or prevent disease progression to MF or Acute Myeloid Leukemia (AML) [63]. Otherwise, DNMT inhibitors, such as azacitidine or decitabine, inhibit DNA methyltransferase enzymes, resulting in the hypomethylation of DNA. This process can reactivate silenced tumor suppressor genes and potentially reverse abnormal epigenetic changes that drive the disease [101].

This is the reason why combinatory treatments can be the driving discovery to more effective therapy. An in-depth analysis is necessary to investigate the potential side effects caused by their combination. For instance, in the case of JAKi combined with DNMTi, possible overlapping toxicities must be considered, because the cytopenia caused by both could limit their combined use.

Moreover, further studies about patients’ genetics are required, to identify those who will benefit most from this combination. At least, ongoing research is evaluating the safety and efficacy of combining these therapies in MPNs. One notable example is the ongoing clinical trial NCT01787487 [102,103], which is evaluating the combination of ruxolitinib with azacitidine in patients with post-MPN MF. Preliminary results indicate that this strategy may enhance disease control, reduce symptom burden, and potentially delay progression. Other trials are investigating HDAC inhibitors and BET inhibitors [104] as adjunct therapies to target inflammatory signaling and clonal expansion—both features potentially exacerbated by the 46/1 haplotype. Most of the available data are preclinical or clinical but lack stratification based on the 46/1 haplotype. Preclinical studies have shown synergy between HDAC and BET inhibition in various cancer types, including acute myeloid leukemia, solid tumors, and medulloblastoma [105]. These studies demonstrate the potential of these inhibitors to reduce inflammatory signaling, suppress MYC, and induce apoptosis [105]. Based on these preclinical findings, combining a BET inhibitor (e.g., pelabresib) with an HDAC inhibitor (e.g., givinostat, panobinostat, or others) may be effective in suppressing JAK2 signaling, particularly in 46/1 carriers. This combination could also lead to reduced inflammation, shrinkage of the spleen, and potentially slow clonal expansion [106]. Preclinical data surrounding pracinostat and pacritinib (a JAK inhibitor) suggests that combining epigenetic and kinase inhibition is beneficial in JAK2 (V617F) settings [107]. This further strengthens the rationale for combining HDAC inhibitors or BET inhibitors with JAK inhibitors.

In Table 2, we reported clinical trials that combined therapies with JAK inhibitors and epigenetic agents in MPNs.

Given the haplotype’s potential involvement in epigenetic dysregulation and immune evasion, future therapies could be designed to specifically target these pathways in G allele carriers. This approach aligns with the broader trend toward personalized medicine in MPNs, where treatment strategies are tailored not only to somatic mutations but also to germline risk factors and chromatin architecture.

Nevertheless, several barriers must be addressed before the 46/1 haplotype can be adopted as a routine biomarker in clinical settings. First, genotyping costs and access to high-throughput sequencing technologies may limit widespread implementation, particularly in low-resource healthcare systems. Second, the predictive utility of the haplotype remains to be validated in large, ethnically diverse cohorts and in prospective, longitudinal studies. Third, while its association with JAK2 V617F and disease phenotypes is well established, its predictive value for treatment response and long-term outcomes needs further investigation.

## 9. Discussion

The JAK2 46/1 haplotype has emerged as a significant germline genetic factor that predisposes individuals to myeloproliferative neoplasms (MPNs), particularly through its strong association with the acquisition of the JAK2 V617F somatic mutation [47,89]. This relationship underscores its potential as a predictive biomarker for MPN onset and progression. Detecting the haplotype in patients may provide clinicians with a tool for early identification of individuals at higher risk of developing MPNs, offering opportunities for surveillance and timely intervention.

Two main hypotheses have been proposed to explain the mechanistic role of the JAK2 46/1 haplotype in MPNs pathogenesis.

The “hypermutability” hypothesis suggests that the JAK2 46/1 haplotype makes the genetic region in which it is located more genetically unstable and more susceptible to replication errors and genetic damage [6,47]. Conversely, the “fertile soil” hypothesis postulates that hematopoietic stem cells (HSCs) carrying the 46/1 haplotype have a selective advantage. This means that these cells might proliferate or survive better than other HSCs, creating a more “fertile” environment for the development of mutations and subsequent clonal expansion [5]. Importantly, these two hypotheses are not mutually exclusive and could both contribute to the risk of MPNs.

In addition to its role in disease initiation, recent evidence implicates the 46/1 haplotype in disease progression, therapy resistance, and clonal evolution [48].

It appears to enhance activation of downstream signaling pathways, including JAK-STAT, MAPK and PI3K/AKT, conferring a survival and proliferative advantage to malignant clones even in the presence of pharmacologic JAK2 inhibition [107]. This suggests a potential role in drug resistance, particularly to JAK inhibitors such as ruxolitinib, which remains a cornerstone of MPNs treatment. Epigenetic dysregulation associated with the haplotype may further sustain these oncogenic transcriptional programs and drug resistance, highlighting the potential benefit of combination therapies incorporating epigenetic modifiers alongside JAK inhibitors [95].

A growing body of data suggests that the 46/1 haplotype may predict the onset of onco-drug resistance in patients with MPN who are positive for the V617F mutation. The onset of resistance to JAK inhibitors, particularly ruxolitinib—currently a mainstay treatment for MPNs—is a significant clinical challenge that undermines long-term disease control and patient survival. Evidence indicates that the presence of the JAK2 46/1 haplotype may contribute to this resistance phenotype. An association has been discovered between patients with the G allele 46/1 haplotype, tagged by the rs10974944 SNP, and the predisposition to the development of clinical parameters that could cause onco-drug resistance [7]. Consequently, it has been supposed that the presence of the 46/1 haplotype predisposes to thrombocytosis, leukocytosis, splenomegaly and hepatomegaly, characteristic symptoms of MPNs, which in turn lead to onco-drug resistance. If this hypothesis is confirmed, the haplotype could become a prognostic marker for the outcome of the disease, by predicting the onset of the onco-drug resistance and guiding medical decisions concerning the therapies.

Furthermore, the G allele of the 46/1 haplotype, tagged by the rs10974944 SNP, has been demonstrated to be associated with the evolution of PV or ET to a myelofibrotic condition [7] reinforcing its potential utility as a prognostic marker.

These results would be useful for medical personnel to predict the rise and evolution of MPNs, guiding them to better treatment, considering the increased probability of evolution to MF in patients with this haplotype.

From a diagnostic standpoint, one of the major advantages of the 46/1 haplotype is its early detectability, even before the acquisition of somatic driver mutations such as V617F.

The haplotype can be identified using simple, cost-effective molecular techniques—such as Polymerase Chain Reaction (PCR) based assay or PCR-Restriction Fragment Length Polymorphism (PCR-RFLP) methods, which are routinely performed in clinical laboratories. Protocols developed by Trifa et al. [108] and subsequently refined by Pagliarini-e-Silva [52] and Perrone et al. [7] provide practical and accessible approaches to genotyping this haplotype using the rs10974944 SNP and others. The availability of multiple tagging SNPs facilitates robust assay design, further supporting its implementation in clinical settings.

Incorporating haplotype screening into early diagnostic workflows could represent a paradigm shift in MPN management—moving from reactive to proactive, genotype-informed clinical decision-making. Given that the 46/1 haplotype precedes V617F acquisition, it could serve as an early warning indicator, especially in patients with familial predisposition or borderline hematologic abnormalities.

However, despite the accumulating evidence, significant gaps remain. The precise molecular mechanisms through which the 46/1 haplotype exerts its effects remain incompletely understood, highlighted by the conflicting evidence.

In fact, while the key role of JH2 domain is well recognized, the specific regulatory elements and epigenetic changes that modulate oncogenic signaling and drug resistance need to be elucidated. Addressing these knowledge gaps will be critical for the development of targeted therapies and improved risk stratification models.

Moreover, a comprehensive epidemiological analysis of the 46/1 haplotype’s prevalence across diverse populations is currently lacking. Studies from Brazil, Europe, and Japan, provide valuable insights, but broader investigations are necessary to understand the ethnic and geographic distribution of the haplotype. Such data could refine our understanding of population specific risk and improve the global applicability of haplotype-based biomarkers.

To sum up, the potential of the haplotype is unmistakable; therefore, further studies are necessary to evaluate its practical application.

## 10. Conclusions

The haplotype 46/1 is one of the major discoveries in hematological malignancy management, to such an extent that it has been proposed as a novel marker for early diagnosis and risk stratification in MPN.

The haplotype 46/1 predisposes MPN patients to the acquisition of the V617F mutation, representing an innovative target for early diagnosis. Moreover, it offers many advantages because it is easily detectable with non-invasive methods and it allows for personalized diagnostic approaches, based on the genetic profile of each patient.

In addition, many studies have demonstrated the role in leading to the onset of onco-drug resistance. This implies that it might be a useful predictive biomarker for an individual’s susceptibility to develop the symptoms typical of this condition.

Nevertheless, this evidence must be validated by further studies, but it represents promising preliminary results for the inclusion of the haplotype in the field of personalized medicine.

## 11. Future Directions

Looking ahead, further investigations are warranted to bridge the existing knowledge gaps. A major limitation in the current literature is the lack of longitudinal data. Consequently, further studies should aim to extend the sample under study, maybe with multicenter research.

Further elucidation of the epigenetic landscape of the haplotype may uncover novel therapeutic targets. Understanding these mechanisms could pave the way for novel diagnostic tools or therapeutic strategies, allowing a precise and quick management of patients with high risk stratification.

Overall, these future directions hold great promise for enhancing our understanding and management of myeloproliferative neoplasms.

## Figures and Tables

**Table 1:** The table summarizes the studies carried out in recent years to highlight the correlation between the 46/1 haplotype and the myeloproliferative neoplasms and the onco-drug resistance onset. MPN= Myeloproliferative Neoplasm, SNP = Single-Nucleotide Polymorphism, JAK2 = Janus kinase 2, PMF = Primary MyeloFibrosis, PD-L1 = Programmed Death-1 receptor Ligand.

Title	Year of Publication	Main Discovery	Limits
A germline JAK2 SNP is associated with predisposition to the development of JAK2 V617F -positive myeloproliferative neoplasms (doi: 10.1038/ng.342)	2009	This study demonstrates that the V617F mutation is preferentially acquired in cis with the 46/1 haplotype, suggesting a key role of the predisposition allele in MPN acquisition.	Further studies are required to confirm the data obtained.
JAK2 haplotype is a major risk factor for the development of myeloproliferative neoplasms (doi: 10.1038/ng.334)	2009	This study highlights the role of the 46/1 haplotype in the predisposition to MPN.	Further studies are required to confirm the data obtained.
JAK2 46/1 haplotype analysis in myeloproliferative neoplasms and acute myeloid leukemia (doi: 10.1038/leu.2010.172)	2010	This study confirms that the haplotype is a predisposing factor for V617F-positive MPNs acquisition. It also consolidates the hypothesis that this mutation preferentially arises on the considered haplotype.	Further studies are required to confirm the data obtained.
Evaluation of the association between the JAK2 46/1 haplotype and chronic myeloproliferative neoplasms in a Brazilian population (doi: 10.6061/clinics/2013(01) oa02)	2013	This study confirms the association between the 46/1 haplotype and BCR/ABL-negative MPNs.	Further studies are needed to investigate the molecular and genetic mechanisms of intracellular signaling involved in this pathway and to identify new biomarkers for diagnostic and therapeutic purposes.
The influence of novel transcriptional regulatory element in intron 14 on the expression of Janus kinase 2 gene in myeloproliferative neoplasms (doi: 10.1007/s13353-012-0125-x)	2013	This study suggests that the haplotype expression does not interfere with Jak2 gene expression in MPN patients.	Further studies are required to confirm the data obtained.
JAK2 46/1 haplotype is associated with JAK2 V617F positive myeloproliferative neoplasms in Brazilian patients (doi: 10.1111/ijlh.12380)	2015	In the present work, the G allele of the 46/1 haplotype has been associated with MPNs, especially in V617F-positive patients and with higher levels of hemoglobin in the Brazilian population.	Further studies are needed to confirm the data obtained and to investigate the epidemiological distribution of the considered variant.
The germline JAK2 GGCC (46/1) haplotype and survival among 414 molecularly annotated patients with primary myelofibrosis (doi: 10.1002/ajh.25349)	2019	This study reveals that nullizygosity for the 46/1 haplotype is associated with inferior survival in patients with JAK2 V617F-positive PMF.	Further studies are needed to confirm the data obtained with a stronger statistical analysis.
Association of JAK2 Haplotype GGCC_46/1 with the Response to Onco-Drug in MPNs Patients Positive for JAK2 V617F Mutation (doi.org/10.3390/onco4030018)	2024	This work highlights that G/G allele is associated with disease progression to myelofibrosis and certain resistance-related clinical parameters.	The narrow cardinality of the sample under study does not allow a significant static correlation to validate the preliminary results obtained.
3D insights: JAK2 46/1 haplotype shapes MPN development (doi: 10.1182/blood.2025028547)	2025	This study investigates the link between immune checkpoint regulation and the 46/1 haplotype. It suggests that an immunosuppressive microenvironment in the bone marrow could be triggered by the presence of MPN-derived stem cells characterized by high PD-L1 expression. This background could stimulate the clonal expansion of cells with JAK2 mutations, explaining the genetic predisposition to MPNs. Moreover, the study highlighted that the physical interaction between PD-L1 and JAK2 changes in patients with the 46/1 haplotype and in those who have the non-risk haplotype.	The unclear mechanism of PD-L1 in MPN pathogenesis must be studied.
The JAK2 46/1 haplotype influences PD-L1 expression (doi: 10.1182/blood.2023023787)	2025	The present study suggests a new mechanism by which the haplotype could predispose to an increased risk of developing MPNs. In fact, it might be influenced by a concomitant higher expression of PD-L1.	Further studies are required to confirm the data obtained.

**Table 2:** The table summarizes clinical trials investigating the combination of JAK inhibitors (mostly ruxolitinib) with epigenetic agents (such as hypomethylating agents or BET inhibitors) in myeloproliferative neoplasms (MPNs). It includes trial identifiers, drug combinations, target patient populations, trial goals, and current status. MPN= Myeloproliferative Neoplasm, MF = Myelofibrosis (includes primary, post-ET, post-PV), MDS/MPN-U = Myelodysplastic/Myeloproliferative Neoplasm—Unclassifiable, MPN-AP/BP = Accelerated or Blast Phase of MPN, DNMT inhibitor = DNA Methyltransferase Inhibitor, HDAC inhibitor = Histone Deacetylase Inhibitor, BET inhibitor = Bromodomain and Extra-Terminal domain inhibitor, ORR = Overall Response Rate, OS = Overall Survival, SVR = Spleen Volume Reduction, Wk = Week, JAKi = JAK Inhibitor.

NCT/Identifier	Epigenetic Agent(s)	JAK Inhibitor(s)	MPN Population/Phase(s)	Key Endpoints / Preliminary Results	Status (Recruiting /Complete/Not Recruiting)
NCT01787487 (doi: 10.1182/blood-2018-04-846626)	*Azacitidine* (hypomethylating agent)	*Ruxolitinib*	Myelofibrosis (PMF, post-PV MF, post-ET MF) & MDS/MPN-U etc. requiring therapy; intermediate/high risk MF etc.	Primary: Objective response rate; also, spleen reduction, improvements in bone marrow fibrosis. Results so far: IWG-MRT responses ~72%; >50% spleen reduction in many; improvements in fibrosis in ~57% at 24 months; some cytopenias as toxicity.	Recruiting; primary completion ~April 2027.
NCT02076191 (doi: 10.1182/bloodadvances.2020002119)	*Decitabine* (hypomethylating agent)	*Ruxolitinib*	MPN-Accelerated Phase/Blast Phase (AP/BP) disease (post-ET, post-PV, or MF)	Primary: response rate (CR, CRi, PR, etc.); median overall survival ~9.5 months; ORR ~44%. Tolerability reasonable.	Completed
--- (doi: 10.1080/10428194.2018.1543876)	*Pracinostat* (HDAC inhibitor)	*Ruxolitinib*	Myelofibrosis	A phase 2 study: 80% had “clinical improvement”, spleen response ~74%, symptom response ~80%, some improvements in fibrosis; toxicity (anemia, etc.) high; frequent dose reductions.	Completed/Not Recruiting
MANIFEST-2 (NCT04603495) (doi: 10.1038/s41591-025-03572-3)	*Pelabresib* (BET inhibitor — epigenetic reader)	*Ruxolitinib*	JAK inhibitor-naive Myelofibrosis patients	Primary endpoint (SVR≥35% at week 24) met: ~65.9% vs. ~35.2% with placebo+ruxolitinib. Also, improvements in symptoms and bone marrow morphology.	Ongoing/Recently reported Phase 3. (JAKi-naive)

## Data Availability

No new data were created or analyzed in this study. Data sharing is not applicable to this article.
